# Formalin-fixed stool improves the performance of the Kato–Katz method

**DOI:** 10.14202/vetworld.2024.99-107

**Published:** 2024-01-08

**Authors:** Ampas Wisetmora, Atchara Artchayasawat, Porntip Laummaunwai, Opal Pitaksakulrat, Oranard Wattanawong, Thidarut Boonmars

**Affiliations:** 1 Department of Parasitology, Faculty of Medicine, Khon Kaen University, Khon Kaen, 40002, Thailand; 2 Division of General Communicable Diseases, Department of Disease Control, Ministry of Public Health, Nonthaburi, 11000, Thailand; 3 Office of Diseases Prevention and Control 4 Saraburi, Ministry of Public Health, Saraburi, 18120, Thailand

**Keywords:** formalin-fixed stool, glycerol, Kato–Katz method, parasitology examination, specimen preparation

## Abstract

**Background and Aim:**

Parasitic infections are a public health problem worldwide, including in Thailand. An epidemiological survey for helminthiasis based on stool examination uses the Kato–Katz method as recommended by the World Health Organization. Limitations of this method include the need for fresh stool, time requirement, and lack of quality control. The aim of this study was to enhance the efficiency of the Kato–Katz technique using formalin and glycerol solutions and to implement specimen preparation in fieldwork.

**Materials and Methods:**

For the Kato–Katz method, stool samples were divided into formalin-fixed and unfixed groups at various time points and processes. Fresh echinostome eggs were added to each stool group. Incubation with glycerol increased the clearing process. Each group was observed and photographed using a light microscope. Parasite eggs were imaged and compared using the standard Kato–Katz method.

**Results:**

Visualization of echinostome eggs from formalin-fixed stool slides was significantly better than that from unfixed stool slides (p < 0.01). Stool samples fixed for 7 days retained normal echinostome eggs morphology. Incubation with glycerol for 1 h resulted in increased Kato–Katz performance by digesting the stool content and enhancing egg observation. Moreover, the results of the Kato–Katz method using fixed and fixed stool plus glycerol for natural helminth infection showed good quality of *Opisthorchis viverrini* and *Taenia* egg visualization and normal morphology with a clear background of slides.

**Conclusion:**

Formalin-fixed stool could be more suitable than fresh stool for the Kato–Katz method.

## Introduction

According to the World Health Organization (WHO), more than 3 billion people worldwide suffer from one or more parasitic diseases. These diseases are widespread and are a leading cause of morbidity and mortality [1]. The WHO classifies parasitic diseases as neglected tropical diseases (forgotten diseases), and helminthiasis is a serious silent threat. In 2010, it was estimated that 819 million people were infected with parasitic worms worldwide. Disability-adjusted life years (DALYs) [2], 26 million people will become infected with helminths and 5.2 million will be infected with soil-transmitted helminths from all tropical countries around the world, including Thailand [3].

In most countries, parasite control programs use fecal samples for parasitological diagnosis. As recommended by the WHO [4], the optimal and standard technique is the Kato–Katz method approach. This technique is simple, inexpensive, and field-applicable. Nevertheless, several studies have demonstrated several limitations, including (i) underestimation and low sensibility of prevalence when the parasitic load is low [5, 6], (ii) problems in detecting hookworm infections because the stool must be prepared immediately [7], and (iii) the clearing step (i.e., glycerin) can render the eggs unrecognizable [8]. Kato–Katz method is currently limited to fresh stool specimens. It requires a long sample preparation period, requires microscopy skills, and is relatively immobile during analysis.

This study aimed to improve the diagnostic performance of the KatoKatz method so that it does not interfere with routine fieldwork. The sample preparation approach was optimized, including variations in the general chemical reagent conditions such as formalin, glycerol, and various pH conditions used in parasitological diagnosis. Egg visualization and characterization were the focus of this study.

## Materials and Methods

### Ethical approval

The Animal Ethics Committee (AEMDKKU0322) and the Human Ethics Committee (HE6510003) of Khon Kaen University approved all parasite specimens, human stool, and protocols.

### Study period and location

The study was conducted from January to November 2022 in the Department of Parasitology, Faculty of Medicine, Khon Kaen University, Thailand.

### Parasite egg preparation

Metacercariae of echinostomes were collected from *Indoplanorbis exustus* snails in Khon Kaen province, infected with 50 metacercariae/hamster, and maintained for approximately 1 month. After 1 month, the hamsters were sacrificed and the small intestines and adult worms were collected for echinostome eggs. Fresh human stool samples were leftover specimens from Srinagarind Hospital. There were two types of echinostome: formalin-fixed echinostome eggs and fresh echinostome eggs.

### General Kato–Katz method

Cellophane strips of the size of a microscope slide were cut and immersed in 3% malachite green solution in glycerol for 24 h before use. We performed the Kato–Katz method following the WHO protocol [9, 10]. In brief, 0.1 g of fresh stool was sieved, covered with cellulose membrane, incubated with malachite green glycerol solution, and observed under a light microscope at 40X magnification. Here, 200 echinostome eggs were added to each stool sample. The egg morphology of each assigned group was recorded as normal or irregular.

### The effect of pH on echinostome egg morphology post-Kato–Katz method

**Figure-1:**
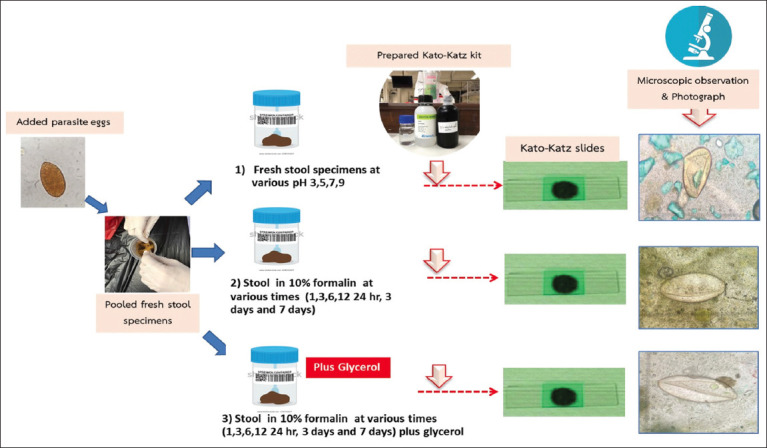
The diagram of all assigned protocols.

A normal saline solution (NSS) at different pH levels (3, 5, 7, and 9) was prepared to assess whether the pH of the solution affected the egg shape of the echinostome. A pH meter and pH indicator strips were used to determine the pH values of the solutions. To observe egg morphological changes on Kato–Katz slides, the prepared solutions were incubated with egg parasites for 1, 3, and 6 h at 25°C (Figure-1). The pH values of the NSS used to prepare the stool specimens were recorded using the Kato–Katz method. The echinostome egg morphology was examined, and the overall area under a light microscope was examined at various times (1, 3, 6 h) and compared with normal egg morphology using a simple smear technique.

### Effect of formalin fixation using the Kato–Katz method on echinostome egg morphology

A 10% formalin solution is commonly used to preserve stool for routine laboratory testing and is recommended for helminth eggs and larvae following the WHO protocol [10] to maintain the egg morphology during the Kato–Katz method preparation. Fresh echinostome eggs were then added to fresh stool and divided into two groups with three replicates: i) fresh stool control and ii) 10% formalin-preserved stool. In brief, the fresh stool group was placed at 25°C, and the preserved stools were fixed with 10% formalin solution at a ratio of 1:1 (10%) to stool for 1, 3, 6, and 12 h, respectively. The assigned stools were processed using the Kato–Kaz method, as shown in Figure-1. In brief, 0.2 g of feces was sieved, covered with cellulose membrane, and incubated with malachite green plus glycerol solution according to the WHO recommendation. We examined the echinostome egg morphology and observed the overall area under a light microscope in the fresh stool group compared with the preserved stool group. The egg morphology was photographed and recorded as normal or irregular.

### Post-Kato–Katz slide visualization enhancement by incubation with glycerol

**Figure-2:**
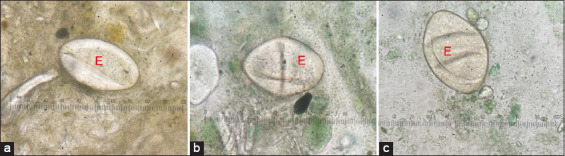
The scoring of Kato–Katz slide, (a) +1: The fecal particles were larger than +2, (b) +2: The fecal particles were smaller than +1, (c) +3: The fecal particles were smaller than +2.

Stools preserved for 1, 3, 6, 12, and 24 h, 3 days, and 7 days were divided into two groups: (i) Formalin preservation with 10% formalin and (ii) formalin preservation and glycerol incubation for 1 h. Subsequently, they were further processed according to the Kato–Katz method, as shown in Figure-1. The 10% formalin to stool volume ratio was 1:1 and the 10% formalin to stool volume to glycerol volume ratio was 1:1:1. In brief, 0.2 g of feces was sieved, covered with cellulose membrane, and then incubated with malachite green plus glycerol solution as described above. The parasite eggs were examined and the egg morphology was observed. The overall area was then visualized under a light microscope within 1 h. As shown in Figure-2, the background of each Kato–Katz slide was recorded as +1 = fecal particles larger than +2, +2 = fecal particles smaller than +1, and +3 = fecal particles smaller than +2. The egg and Kato–Katz slides were photographed and recorded as normal or irregular morphology.

### Implementation of formalin-fixed stool improves Kato–Katz method performance for natural helminth infections

One hundred and twenty-five stool specimens from Srinagarind Hospital were randomly collected and processed according to the Kato–Katz method (November 2022) in three groups: unfixed, fixed, and fixed stool plus glycerol groups. Positive slides were then visualized under a light microscope within 1 h. Only the *Opisthorchis viverrini* (OV) slides were observed at 9, 24, 36, and 48 h. The background of each Kato–Katz slide was recorded as follows: +1 = Fecal particles larger than +2, +2 = Fecal particles smaller than +1. Egg morphology and the Kato–Katz slides were photographed and recorded as normal or irregular.

### Statistical analysis

A statistically significant number of parasite eggs were found in all stool samples. The number of stool samples that tested positive for parasites was determined. Frequencies and percentages were used to summarize categorical variables, such as microscopy positivity and smear efficiency, and were analyzed by comparing means using the paired samples t-test, two independent groups of data. Statistically significant differences were considered when p = 0.05 (p < 0.05). The Statistical Package for the Social Sciences version 25 software (IBM Corp., NY, USA) was used for statistical analysis.

## Results

### Effect of pH on microscopic egg morphology using the Kato–Katz method

**Figure-3:**
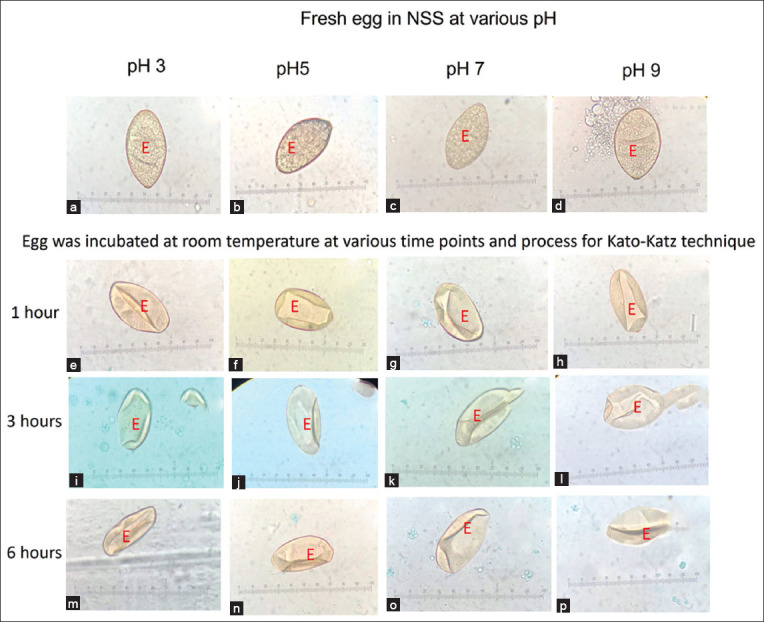
(a-p) The appearance of echinostome egg in various pH 3, 5, 7, and 9 using simple smear and Kato–Katz technique, respectively.

**Figure-4:**
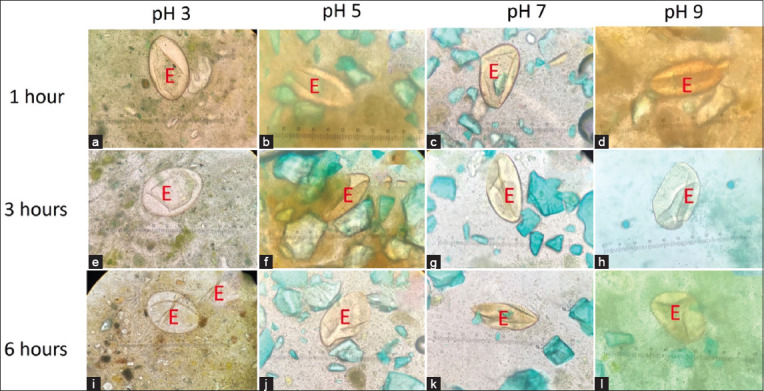
(a-l) The appearance of echinostome egg from Kato–Katz slide using unfixed stool at various time points; Egfg (E).

The egg morphology at 6 h in NSS was similar at every pH (Figure-3). However, the morphological changes of the eggs from the Kato–Katz slides in all pH groups exhibited the same patterns, including folds in the eggshells, irregular egg shapes, and surface clearing. However, fresh stool obtained using the Kato–Katz method showed results similar to those obtained with the egg morphology without feces described above (Figure-4). Interestingly, some of the echinostome eggs were digested and had an abnormal morphology, that is, enlarged size and malformed shape, which is difficult to differentiate.

### Use of formalin-fixed stool for the Kato–Katz method during the COVID-19 pandemic in a field study

**Figure-5:**
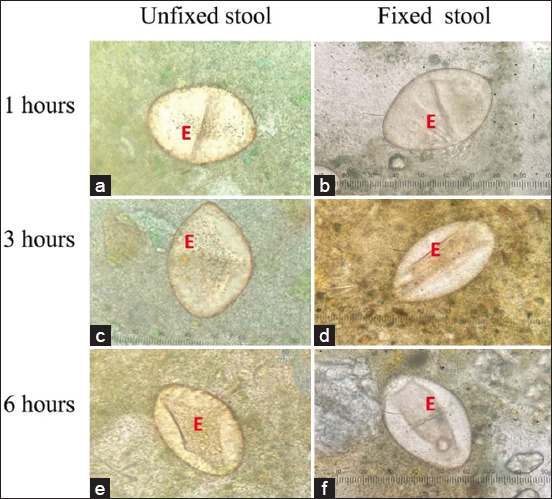
The appearance of echinostome egg from Kato–Katz slide using (a-c) unfixed stool and (d-f) formalin-preserved stool at 1, 3, and 6 h; Egg (E).

Echinostome eggs were observed at the 1^st^, 3^rd^, and 6^th^ h from the Kato–Katz slides using unfixed and formalin-preserved stool samples. The eggs had a very good eggshell shape, most of them had organelles, especially in the preserved stool (Figure-5e and f). Eggshell swelling was observed in unfixed and preserved stool samples (Figure-5a, b, c and d). The unfixed stool was fermented at 25°C for 12 h, and the strong smell stopped the experiment.

### Enhancing the visualization of parasite eggs using the Kato–Katz method by glycerol incubation before processing

**Figure-6:**
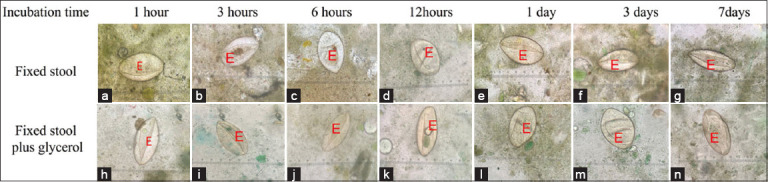
The appearance of echinostome egg and stool particle of Kato–Katz slide using (a-g) formalin-preserved stool and (h–n) formalin-preserved plus glycerol; Egg (E).

This study aimed to determine whether the glycerol solution could digest formalin-fixed stool at various time points of formalin fixation (1, 3, 6, 12, 24 h, 3 days, and 7 days). Both fixed stools and fixed stools plus glycerol had echinostome eggs post-Kato–Katz processing (Figure-6). However, formalin fixation plus glycerol (Figure-6h-n) groups were finer and more easily observed than formalin fixation alone (Figure-6a-g) groups. The particles in the stool sample were digested to a small size; the egg shapes and backgrounds were very clear.

Table-1 summarizes all testing periods and degradation of fecal fibers in the formalin-preserved groups. The fecal fibers were finer and more digestible in the formalin groups, and the clarity of the fecal slide was significantly better than that of the fresh fecal slide (95% confidence interval [CI]: −0.856 to −0.462, p = 0.000). The group preserved with formalin and glycerol had finer and more digestible fecal fibers. The clarity of the fecal slide was significantly better than that of the fresh fecal slide (95% CI: −1.371 to −0.940, p = 0.000). Samples preserved with formalin and glycerol were significantly better digested and the clarity of the fecal slide was significantly better than that of samples preserved with formalin alone (95% CI: −0.723 to −0.271; p = 0.000).

**Table-1:** A visualization and clarification of Kato–Katz slide on experiment groups.

Groups	Mean	SD	95% CI		t	df	p-value

			Lower	Upper			
Unfixed stool specimens-Formalin preservation	–0.659	1.288	–0.856	–0.462	–6.607	166	0.000
Unfixed stool specimens-Formalin and glycerol preservation	–1.156	1.410	–1.371	–0.940	–10.593	166	0.000
Formalin preservation-Formalin and glycerol preservation	–0.497	1.476	–0.723	–0.271	–4.351	166	0.000

SD=Standard deviation, CI=Confidence interval

The formalin-preserved group had no statistically significant difference in the degradability of fecal fibers at the 1^st^, 3^rd^, and 6^th^ periods compared with the control fresh fecal sample (Table-2). The formalin- and glycerol-preserved group was compared with the fresh fecal control sample and the formalin-preserved group at the 1^st^, 3^rd^, and 6^th^ h; fixation was significantly more effective over time. The clarity and degradation of the fecal fibers were finer and more digestible, and the clarity of the fecal slide was significantly improved. The group was preserved with formalin and glycerol except on day 7. Degradation and fineness of the fibers were not statistically different compared with those preserved with formalin (Table-2).

**Table-2:** A comparison of visualization and clarification of Kato–Katz slide at various time points.

Groups	Mean	SD	95% CI		t	df	p-value

			Lower	Upper			
Unfixed stool specimens-Formalin preservation (1 h)	0.027	0.164	–0.028	0.082	1.000	36	0.324
Unfixed stool specimens-Formalin and glycerol preservation (1 h)	–1.640	0.490	–1.842	–1.438	–16.738	24	0.000
Formalin preservation-Formalin and glycerol preservation (1 h)	–1.640	0.490	–1.842	–1.438	–16.738	24	0.000
Unfixed stool specimens/Formalin preservation (3 h)	–0.043	0.209	–0.134	0.047	–1.000	22	0.328
Unfixed stool specimens-Formalin and glycerol preservation (3 h)	–1.750	0.447	–1.988	–1.512	–15.652	15	0.000
Formalin preservation-Formalin and glycerol preservation (3 h)	–1.750	0.447	–1.988	–1.512	–15.652	15	0.000
Unfixed stool specimens-formalin preservation (6 h)	–0.188	0.403	–0.402	0.027	–1.861	15	0.083
Unfixed stool specimens-Formalin and glycerol preservation (6 h)	–1.727	0.905	–2.335	–1.120	–6.333	10	0.000
Formalin preservation-Formalin and glycerol preservation (6 h)	–1.545	1.214	–2.361	–0.730	–4.224	10	0.002
Formalin preservation-Formalin and glycerol preservation (12 h)	–1.923	0.494	–2.221	–1.625	–14.049	12	0.000
Formalin preservation-Formalin and glycerol preservation (24 h)	–1.105	0.459	–1.326	–0.884	–10.500	18	0.000
Formalin preservation-formalin and glycerol preservation (3 days)	–0.500	0.527	–0.877	–0.123	–3.000	9	0.015
Formalin preservation-Formalin and glycerol preservation (7 days)	0.158	0.688	–1.174	0.490	1.000	18	0.331

SD=Standard deviation, CI=Confidence interval

### Formalin-fixed stool improves Kato–Katz method performance for natural helminth infections

**Figure-7:**
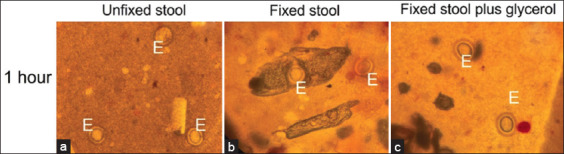
The appearance of *Taenia* egg and stool particle of Kato–Katz slide at 1^st^ h using (a) unfixed stool**,** (b) formalin-preserved stool, and (c) formalin-preserved plus glycerol; Egg (E).

**Figure-8:**
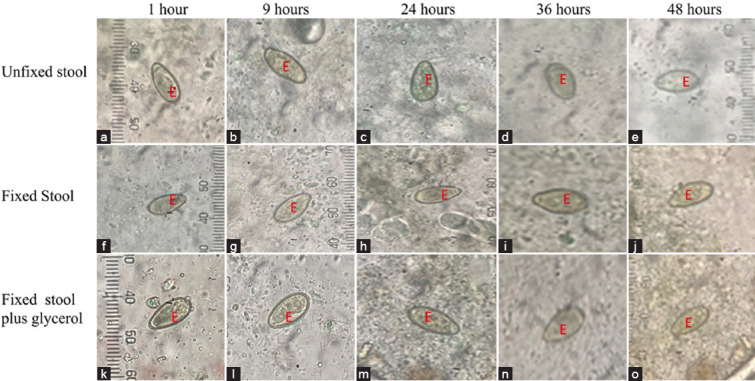
The appearance of *Opisthorchis viverrini* egg and stool particle of Kato–Katz slide at various time point (1^st^, 9^th^, 24^th^, 36^th^, and 48^th^ h) using unfixed stool (a-e) formalin-preserved stool (f-j) and formalin-preserved plus glycerol (k-o); Egg (E).

Five out of 125 stool specimens were positive for helminths. There were three cases of OV and two cases of *Taenia* in all three groups of experiments. The morphology of *Taenia* (Figure-7) and OV (Figure-8) in all three types of experiments was normal at the 1^st^ h with different background of slides. The slide background of fixed stool plus glycerol was clearer than that of the other groups, similar to that observed in the above experiments. We found that all groups (unfixed, fixed, and fixed stool plus glycerol) had OV eggs post-Kato–Katz processing (Figure-8). However, at 1^st^ and 9^th^ h, formalin fixation plus glycerol (Figure-8k-o) groups were finer and more easily observed than unfixed (Figure-8a-e) and fixed stool (Figure-8f-j) groups. The clarity of the fecal slide was significantly better than that of the unfixed stool, as shown in Table-3. The particles in the stool sample were digested to a small size; the egg shapes and backgrounds were very clear. After more than 24 h of observation, the OV eggs of the unfixed stool were significantly degraded with irregular morphology and were difficult to distinguish (Figure-8c-e and Tables-3, 4). At the 36^th^–48^th^ h, both fixed and fixed plus glycerol stool slides were dried, and the OV egg morphology remained in a normal shape.

**Table-3:** A comparison of morphological OV egg of Kato–Katz slide at various time points.

Groups	Mean	SD	95% CI		p-value

			Lower	Upper	
Unfixed stool specimens-Formalin preservation (1 h)	–0.008	0.100	–0.211	0.196	0.940
Unfixed stool specimens-Formalin and glycerol preservation (1 h)	0.083	0.098	–0.115	0.282	0.399
Formalin preservation-Formalin and glycerol preservation (1 h)	0.091	0.100	–0.112	0.294	0.369
Unfixed stool specimens-Formalin preservation (9 h)	0.242	0.140	–0.042	0.527	0.092
Unfixed stool specimens-Formalin and glycerol preservation (9 h)	0.333	0.136	0.055	0.611	0.020
Formalin preservation-Formalin and glycerol preservation (9 h)	0.091	0.140	–0.193	0.375	0.519
Unfixed stool specimens-Formalin preservation (24 h)	0.326	0.144	0.032	0.620	0.031
Unfixed stool specimens-Formalin and glycerol preservation (24 h)	0.417	0.141	0.129	0.704	0.006
Formalin preservation-Formalin and glycerol preservation (24 h)	0.091	0.144	–0.203	0.385	0.533
Unfixed stool specimens-Formalin preservation (36 h)	0.492	0.144	0.198	0.786	0.002
Unfixed stool specimens-Formalin and glycerol preservation (36 h)	0.583	0.141	0.296	0.871	0.000
Formalin preservation-Formalin and glycerol preservation (36 h)	0.091	0.144	–0.203	0.385	0.533
Unfixed stool specimens-Formalin preservation (48 h)	0.492	0.144	0.198	0.786	0.002
Unfixed stool specimens-Formalin and glycerol preservation (48 h)	0.583	0.141	0.296	0.871	0.000
Formalin preservation-Formalin and glycerol preservation (48 h)	0.091	0.144	–0.203	0.385	0.533

OV=*Opisthorchis viverrini*, SD=Standard deviation, CI=Confidence interval

**Table-4:** A comparison of degraded OV egg of Kato–Katz slide at various time points.

Groups	Mean	SD	95% CI		p-value

			Lower	Upper	
Unfixed stool specimens-Formalin preservation (1 h)	–0.008	0.100	–0.211	0.196	0.940
Unfixed stool specimens-Formalin and glycerol preservation (1 h)	0.083	0.098	–0.115	0.282	0.399
Formalin preservation-Formalin and glycerol preservation (1 h)	0.091	0.100	–0.112	0.294	0.369
Unfixed stool specimens-Formalin preservation (9 h)	0.159	0.131	–0.108	0.426	0.234
Unfixed stool specimens-Formalin and glycerol preservation (9 h)	0.250	0.128	–0.011	0.511	0.060
Formalin preservation-Formalin and glycerol preservation (9 h)	0.091	0.131	–0.176	0.358	0.493
Unfixed stool specimens-Formalin preservation (24 h)	0.242	0.140	–0.042	0.527	0.092
Unfixed stool specimens-Formalin and glycerol preservation (24 h)	0.333	0.136	0.055	0.611	0.020
Formalin preservation-Formalin and glycerol preservation (24 h)	0.091	0.140	–0.193	0.375	0.519
Unfixed stool specimens-Formalin preservation (36 h)	0.492	0.161	0.165	0.820	0.004
Unfixed stool specimens-Formalin and glycerol preservation (36 h)	0.500	0.157	0.180	0.820	0.003
Formalin preservation-Formalin and glycerol preservation (36 h)	0.008	0.161	–0.320	0.335	0.963
Unfixed stool specimens-Formalin preservation (48 h)	0.576	0.169	0.232	0.920	0.002
Unfixed stool specimens-Formalin and glycerol preservation (48 h)	0.500	0.165	0.163	0.837	0.005
Formalin preservation-Formalin and glycerol preservation (48 h)	–0.076	0.169	–0.420	0.268	0.657

OV=*Opisthorchis viverrini*, SD=Standard deviation, CI=Confidence interval

## Discussion

The Kato–Katz method is the most commonly used diagnostic approach for helminths, epidemiology studies, and treatment evaluation. As recommended by the WHO [9, 10], the Kato–Katz method shows good performance, particularly at detecting helminth infections of moderate and heavy intensity. Glycerol can digest the eggshell [11]. It is simple and relatively low cost but has a short time window for sample analysis due to stool fermentation and parasite degradation. Post-Kato–Katz method, pH did not affect the shape of the echinostome eggs. The duration of observation was limited to 1 h because more eggshells were cleared; therefore, it was difficult to see and identify hookworms, echinostomes, and/or thin eggshells [7]. We did not observe protozoa cysts or oocytes. Misdiagnosis of other helminths such as *Ascaris*, *Trichuris*, *Schistosoma mansoni*, and hookworm eggs can be misdiagnosed, false positive, or false negative [12–14]. These results suggest the need to examine multiple stool samples from the participants [1, 15].

We found that the stool had a fermented and strong smell approximately 12 h after collection. Fermentation of stool and degradation of helminth eggs affects the diagnostic results, as described by Dacombe *et al*. [16], who reported a decrease in sensitivity of almost 50% for the detection of hookworm with either method when preservation/refrigeration was delayed by more than 3 h [17]. They also reported that after 24 h, mean hookworm FECs dropped close to zero regardless of the storage condition. Whole stool samples stored at 25°C for 1 day resulted in a 23% (p < 0.0001) and 13% (p < 0.0001) decrease in hookworm FEC, respectively. However, the previous reports have found that both debris particles and egg morphological changes can be misdiagnosed [15, 18, 19]. Therefore, false-positive Kato–Katz thick smears where several eggs were counted might mistakenly point to another source of error (e.g., writing errors on the entry forms or eggs confused with eggs from different species) [15]. Figure-2 shows some debris that might resemble an echinostome egg. Similar to false-positive results, false-negative Kato–Katz thick smears that contained a larger number of eggs were probably due to technical skill [20]. The previous reports have shown that readers might be tired due to the high number of thick Kato–Katz smears read per day [21–23]. Therefore, a single Kato–Katz thick smear has low sensitivity, and the proportion of false-negative diagnoses is usually assessed [24–28]. This study was not conducted within the current quality control investigation, so the true number of false-negative samples is likely underestimated [15, 29].

The advantage of using the same slide is that it can still be read up to 48 h later and re-examined, which is not the case with normal Kato–Katz thick smears that must be read within 30–60 min. This is particularly important for hookworm eggs, which disappear over time. In addition, *Ancylostoma duodenale* and *Necator americanus* eggs cannot be morphologically differentiated, making species identification impossible [7].

The previous studies have shown that Kato–Katz with Mini-FLOTAC fixed with 5% formalin [30] and Mini-FLOTAC fixed with sodium acetate fixative (SAF) [31] improved the qualitative diagnosis. Moreover, in agreement with Fernández-Niño *et al*. [32], who used Kato–Katz fixed with SAF, our present study using Kato–Katz fixed with 10% formalin improved the diagnosis.

In the present study, the limitation of stool samples used for testing the Kato–Katz test was the volume and number of stool samples. In our laboratory, echinostomes can be easily maintained using laboratory animals. In the present study, we used a representative egg that shares similarities with hookworm egg which is known for its thin eggshell. However, the culture of hookworm eggs has certain limitations. Therefore, healthy feces mixed with laboratory-collected echinostome eggs were used in this study. This method has been shown to improve the performance of the Kato–Katz method as a reflection of the quality of parasite egg visualization and morphology with a slide background.

## Conclusion

Formalin-preserved stool is useful for the Kato–Katz method, as evidenced by the good morphology of helminth eggs (Echinostome, OV, and *Taenia*). Stool incubation with glycerol before the Kato–Katz process could reduce the background of stool particles by reducing the size of the particles and/or clearing the fiber. Formalin-preserved stools and glycerol incubation enhance Kato–Katz performance. Formalin fixation preserves the fecal sample for future qualitative studies.

## Authors’ Contributions

AW, AA, PL, OP, OW, and TB: Conceptualization, methodology, investigation, and data curation. AW, AA, TB, and OW: Drafted and revised the manuscript. All authors have read, reviewed, and approved the final manuscript.
